# Function predicts how people treat their dogs in a global sample

**DOI:** 10.1038/s41598-023-31938-5

**Published:** 2023-03-27

**Authors:** Angela M. Chira, Kathryn Kirby, Theresa Epperlein, Juliane Bräuer

**Affiliations:** 1 Department of Linguistic and Cultural Evolution, Max Planck Institute for Evolutionary Anthropology, Deutscher Platz 6, 04103 Leipzig, Germany; 2 Max Planck Institute for Geoanthropology, DogStudies, Kahlaische Strasse 10, 07745 Jena, Germany; 3 Department of Ecology and Evolutionary Biology, University of Toronto, Toronto, Canada; 4 Department for General Psychology and Cognitive Neuroscience, Friedrich Schiller University of Jena, Am Steiger 3, 07743 Jena, Germany

**Keywords:** Evolution, Anthropology, Biological anthropology

## Abstract

Dogs have an extraordinary relationship with humans. We understand, communicate, and cooperate remarkably with our dogs. But almost all we know about dog-human bonds, dog behaviour, and dog cognition is limited to Western, Educated, Industrialized, Rich, Democratic (WEIRD) societies. WEIRD dogs are kept for a variety of functions, and these can influence their relationship with their owner, as well as their behaviour and performance in problem-solving tasks. But are such associations representative worldwide? Here we address this by collecting data on the function and perception of dogs in 124 globally distributed societies using the eHRAF cross-cultural database. We hypothesize that keeping dogs for multiple purposes and/or employing dogs for highly cooperative or high investment functions (e.g., herding, guarding of herds, hunting) will lead to closer dog-human bonds: increased primary caregiving (or positive care), decreased negative treatment, and attributing personhood to dogs. Our results show that indeed, the number of functions associates positively with close dog-human interactions. Further, we find increased odds of positive care in societies that use herding dogs (an effect not replicated for hunting), and increased odds of dog personhood in cultures that keep dogs for hunting. Unexpectedly, we see a substantial decrease of dog negative treatment in societies that use watchdogs. Overall, our study shows the mechanistic link between function and the characteristics of dog-human bonds in a global sample. These results are a first step towards challenging the notion that all dogs are the same, and open questions about how function and associated cultural correlates could fuel departures from the ‘typical’ behaviour and social-cognitive skills we commonly associate with our canine friends.

## Introduction

Dogs (*Canis lupus familiaris*) are special to us. For five reasons they are different from all other species: First, they were domesticated earlier than any other animals. Although it is not exactly known when, where and how the dog domestication process started^1,2^, it is clear that it happened before humans settled down, and long before other animals such as sheep (*Ovis aries*) were domesticated^3^. Second, in contrast to species kept for meat, milk and skin—i.e., to feed and dress humans, the reasons why dogs were initially domesticated are still debated^1,4^. It is likely that they were valuable to humans not for a single purpose^5^, and more importantly, their function often involved some cooperation with humans (be it for hunting or guarding^6^). Also nowadays, dogs are kept for various different functions including protection, hunting, herding, but also for rescue, search, service and guide purposes^7–9^. Third, and also in contrast to sheep, goats (*Capra aegagrus hircus*) and cattle (*Bos taurus*), it is hypothesized that dogs travelled a commensal pathway into domestication. That means, in line with the so-called self-domestication hypothesis, dogs` ancestors—wolves (*Canis lupus*)—lived in close proximity to humans. While adapting to the human, these animals had the advantage to exploit new and reliable food sources^4,10^.

Fourth, as dogs have adapted to the human environment, they developed remarkable social-cognitive skills in communication, cooperation, and sensitivity to human behaviour^4,8^. But there are important differences between dogs’ behaviour and performance in various tasks, and some of this variation is most likely related to the function of dogs—established by selection of particular dogs and/or training. However, the links between dog behavioural tendencies, modern breeds, and dog historical working roles are unclear^11^. In a recent study, Dutrow et al.^12^ identify behavioural correlates for eight major dog genetic lineages that correspond to historical purposes for keeping and breeding dogs. In comparison, Morrill et al.^13^ find that breed ancestry is a poor predictor of dog behavioural traits, and the behaviour of individuals dogs varies greatly within breeds. In parallel, research within the framework of animal cognition explore how the purpose for which dogs are kept links with their abilities and behaviour when solving problems. For example, it was found that dogs with a working relationship (dogs kept outside the house as watchdogs or for other purposes) with their owners performed better in a problem-solving task than closely attached family dogs^14^. In more recent studies, the unsolvable-problem-paradigm was used to investigate the relationship between dogs and their owners. In this paradigm, dogs subjects are tasked with obtaining a food reward from a box with a lid. After repeated successes, the animals are confronted with an unsolvable version of the task: the lid is closed and dogs cannot push it to the side anymore to reach the reward^15^. Dogs do not only vary greatly in their persistence in trying to open the box, but also in their motivation to look back to the human standing next to it, presumably seeking for help (but see^16^ for a different explanation). Dogs used for various functions have been tested in this paradigm. Man-trailer dogs were most persistent in this task and worked independently, whereas assistant dogs and water rescue dogs often looked back at person in the room. For agility dogs and family dogs it also mattered who this person was—dogs preferred to look to the owner when the problem became unsolvable^17–20^. Thus, the function of these dogs had an influence on their relationship to their owner, and further, function and dog-owner relationship impacted on dog behaviour and performance in various problem-solving tasks.

But there is an important gap in our knowledge about the dog-human relationship. Although it has been very well investigated in western countries in the last 20 years, we do not know much about this topic in non-western cultures. Nearly all studies about dog-human relationships tested dogs and owners from ‘WEIRD’ societies, i.e., Western, Educated, Industrialized, Rich, Democratic societies^21^. However, most dogs in our world—an estimate of 75%,^4,22^—are not kept in ways that mirror the circumstances in western countries^4,22^. So, it is unclear whether our knowledge on the dog–human bond in WEIRD societies is representative worldwide. There is a recent body of research on the behaviour of free-ranging dog populations in Morocco and India. For example, researchers found a low persistence of Moroccan free-ranging dogs in the unsolvable-problem experiments compared to captive and pet dogs, likely because free-ranging dogs lack experience of human-mediated object interaction^23^. Work on free-ranging dogs has also been used to understand competing hypotheses about why dogs look back at the human experimenter when trying to obtain the food reward^16,24^ (i.e., social problem-solving strategy versus simply looking at the human as the most salient stimulus in the environment). Several studies explored the behaviour of free-ranging dogs in India. Dogs were scored on their ability to obtain food from a puzzle-box^25^, follow human pointing cues^26,27^, respond to the human gaze^28^ and social petting^27,29^). It was concluded that dogs in these populations learn to assess human intentions and default to distrust for humans. However, dogs also adjust their responses based on the social interactions with (and reliability of) the human experimenter. There have been a few ethnographic studies that describe how dogs are kept, treated and perceived in non-western cultures (e.g.,^30–33^). Notably, Gray and Young^22^ surveyed typical pet-human dynamics in 60 different societies. They reported a general human preference to keep young pets, particularly dogs and cats. However, they also highlighted striking differences across the world in terms of feeding and sleeping patterns of dogs, as well as in terms of positive and negative dog-human interactions. Koster^34^ also found considerable cross-cultural variation in the training and care of dogs in the Lowland Neotropics. Recently, Chambers et al.^33^ tested several ecological and cultural correlates for dog-human mutual utility in 144 societies. They concluded that dog-human coevolution was enhanced by a subsistence mode of cooperative hunting and by resource defence. All in all, these studies highlight remarkable differences between cultures in how dogs are kept, treated and perceived. Further, the literature of dog studies also marks that, around the world, dogs are kept for a variety of purposes that differ in the extent to which cooperation with humans is required^7,9^. We know that the function of dogs can impact dog-owner relationship in WEIRD dogs. Are these findings representative worldwide? Specifically, are function and dog-owner relationship linked in a global sample? And if so, would this association translate into global differences in dog behaviour and performance in problem-solving tasks (similar to the results in WEIRD dogs)? Answering these questions represents a first step into understanding whether the cognitive and social skills associated with (WEIRD) dogs are universal versus influenced by the cultural environment the dogs live in^35^.

The aim of this project is to address this knowledge gap by exploring the role of dog function in driving global cultural differences in dog-human relationships. We focus on positive and negative dimensions of dog-human relationships that are relevant for dogs functioning: (i) the extent to which humans provide positive care (i.e., primary caregiving), (ii) the extent to which dogs are perceived as people (i.e., dog personhood), and (iii) evidence of negative attitudes and treatment of dogs. We predict that dog-human relationships are closer (illustrated by more positive care/ less negative treatment/more personhood) when dogs fill more roles in societies. This prediction stems from the intuitive assumption that the perceived and realized value of dogs increases with number of functions. Further, we make predictions for individual dog roles based on the potential cost of keeping dogs, as well as based on dog-owner cooperation levels. We expect the dog-human relationship to be closer where the dominant dog function requires intense training (costly) and cooperation between human and dogs. Accordingly, we predict more positive care and personhood, and less negative treatment in societies that keep dogs for hunting and herding, compared to when dogs are used for guarding. We do note though, that training levels in hunting dogs can vary. For example, in the Neotropics, unexperienced dogs are simply brought on the hunt, and their success depends on their natural propensities and ability to learn from experienced dogs^34^. The cooperation levels between individual dogs and their owner are also minimal in this case. We also note that keeping dogs for guarding herds requires a high investment, as these dogs are typically raised with their flock and are usually large (i.e., consume a lot)^36^. Additionally, dogs’ general utility for humans has been linked with subsistence and ecological constraints^33^, and thus we include these covariates in our models (details in Results section). Lastly, Chambers^33^ speculated that pastoral societies employ dogs to guard the herds, which would remove dogs from close contact with humans and constrain their broader use. We incorporate this hypothesis in our model by including reliance on animal husbandry alongside function of dogs in our models.

## Results

To test our hypotheses, we gather general information about the function and perception of dogs in 124 globally distributed societies (Supplementary Fig. 1, Supplementary Dataset 1). The selection of societies was based on the Standard Cross-Cultural Sample (SCCS)—a sample designed to include societies that shared little contact among them through-out their history. Due to their relatively independent development, the SCCS societies allow for meaningful cross-cultural analyses, and are often used by scholars in the field. We collected our data on dog-human interactions using the eHRAF cross-cultural database (https://ehrafworldcultures.yale.edu)—a free database of ethnographic documents that cover various aspects of cultural and social life. Each paragraph in each ethnography is tagged with the subjects it discusses, e.g., “Domestic animals”. The ethnographies are fully digitized, which allows a precise search for keywords as well as concepts.

For each society, we record information about the presence and absence of a wide variety of dog functions: hunting, defence (i.e., whether societies use watchdogs to guard everything except herds), guarding of herds, herding, using dogs as carry animals (for pulling the sled or as pack animals), as playthings/pets (i.e., for playing, companionship, enjoyment), as a status symbol, for vermin/waste removal, keeping assistance dogs (e.g., for people with disabilities), rescue dogs, and keeping dogs for food. We also record the mentions of stray dogs (i.e., unowned dogs). Additional to presence and absence, each function can also be coded as NA (missing information, i.e., the function is neither mentioned as present/absent, nor there is enough information in the ethnographies to infer the function as present/absent). The data available for each of these functions vary greatly (Supplementary Fig. 2). Many of the functions we surveyed seem minor, and thus we identify five functions to use in our analyses: hunting, defence, guarding of herds, herding, and using dogs as carry animals. These main functions are consistently mentioned in ethnographies (less than 20% NA codes), and they also show a balance of presence vs. absence (i.e., no more than 90% or less than 10% presence or absence codes) to satisfy the rigors of our statistical models. We focus and refer to the main functions only in all our downstream analyses and text.

We further coded three dimensions that describe how people treat their dogs: positive care, negative treatment, and personhood. Societies are marked as showing positive care for their dogs if any of the following are recorded in the ethnographies: dogs are fed with human food, breastfed, allowed to sleep indoors, allowed indoors, groomed, played with, provided with puppy care (i.e., there is intentional raising of puppies), provided with healthcare, provided with special equipment designed for their care, or trained (Supplementary Fig. 3a). Second, we mark societies as showing negative treatment towards their dogs if dogs are not fed, not provided healthcare, dogs are physically abused, or killed as part of regular culling acts, in disagreements with humans, or as a result of an aggressive attitude towards dogs in societies (Supplementary Fig. 3b). Lastly, societies are coded as attributing personhood to their dogs if dogs are given names, talked to, buried, mourned, or there are mentions of dogs being treated as kin or close friends, in distinction to other animals (Supplementary Fig. 3c). Similar to the coding of dog functions, each variable that makes up the dimensions of dog-human relationships is coded as present, absent, or NA.

**Figure 1:**
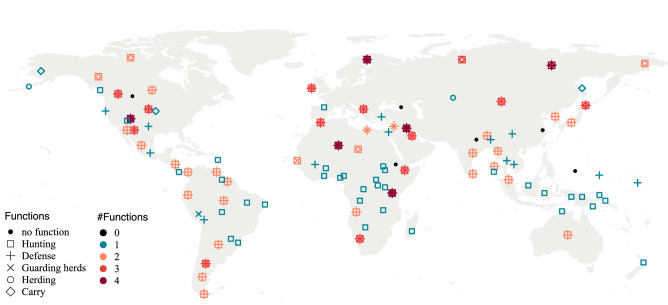
Map of 108 societies with no missing information across at least one of the main functions (hunting, defence, guarding herds, herding, and carry). Colours indicate the number of roles dogs fill in each society, while shapes show what those roles are. This map was generated using the function map (database choice: “world”) within the maps^63^ package in the software R^59^ (version 4.2.2, https://www.R-project.org/).

In agreement with previous literature, we find that dog functions vary greatly across our world (Fig. 1). Out of the initial 124 societies, 108 show non-NA codes (no missing information) for at least one main function (Fig. 1). Interestingly, out of these, 54 (i.e., half of) societies keep dogs for a single purpose, 48 societies show multiple dog functions, and six societies show the absence of the five main functions we consider (Fig. 1). That is, we see variation in the number of roles dogs fill in societies. Hunting was the most widely encountered function (81 societies), followed by defence (55 societies), whereas guarding herds, herding, and carry were not frequent (Supplementary Fig. 2). Similarly, we find that how dogs are perceived and treated also varies globally (Supplementary Fig. 3). In our sample, 77 societies have non-missing information for all three dimensions of dog-human relationships, and 21 societies show the presence of positive care, negative treatment, and personhood. Strikingly, 32 out of the 77 societies with full data show the presence of both positive care and negative treatment, showing that the two axes of dog-human relationships are not mutually exclusive.

### Treatment of dogs and dog functions

**Figure 2:**
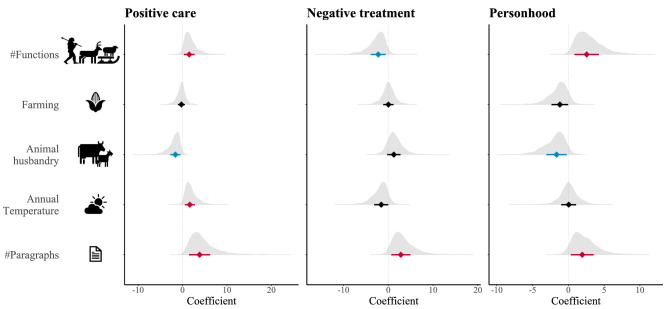
The effect of number of functions dogs fill in societies on the characteristics of dog-human relationships. Predictors for which 95% of the posterior distribution of coefficients is negative or positive are highlighted in blue and red, respectively.

**Figure 3:**
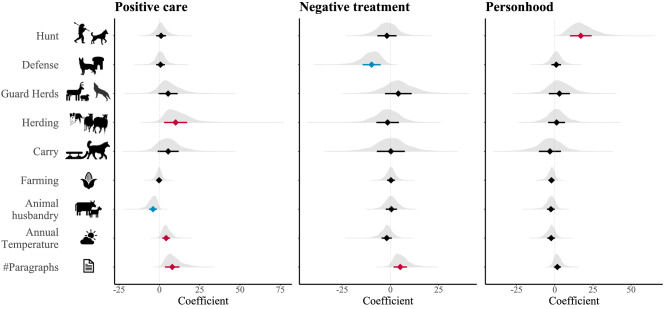
The effect of dog roles (functions) on the characteristics of dog-human relationships. Predictors for which 95% of the posterior distribution of coefficients is negative or positive are highlighted in blue and red, respectively.

To determine the influence of function on dog treatment, we first pruned our data for societies in which there is no missing information for all five main functions (hunting, defence, guarding herds, herding, and carry, a total of 97 societies). We used this pruned dataset to count the number of roles dogs fill in each society. To test our predictions, we first investigate the relationship between the total number of roles dogs fill in societies and their treatment. Second, we look at how the presence or absence of each function impacts the treatment of dogs. We run separate models where the response variable is represented by the presence/absence of positive care, negative treatment, and personhood, respectively. We use binomial Bayesian regressions, and evaluated the coefficient estimates associated with the number of functions, and the presence of each function individually. A positive coefficient value would mean that the target predictor increases odds for the response variable, and the reverse is true for negative coefficients. The Bayesian approach gives us a posterior distribution for these coefficients, which we plot and analyse in its entirety (Figs. 2, 3). We consider a predictor to have substantial effects on the response variable if 95% or more of the posterior distribution of coefficient values is below (for decreased odds) or above (for increased odds) 0.

Subsistence economy, reliance on animal husbandry, as well as environmental variables have been hypothesized to influence dog-human coevolution (e.g.,^33^), hence we account for these parameters in our models. For each society, we use data from the D-PLACE database^37^ to compute farming propensity—a variable capturing a continuum from reliance on hunting, fishing, and gathering to agriculture (Supplementary Fig. 4, see methods for details). We then gather information on reliance on animal husbandry (Supplementary Fig. 5), as well as mean annual temperature at the location of societies. For each society, we also count the number of paragraphs that record information about dogs in ethnographies, as with more paragraphs we expect to see a higher likelihood of rare behaviours being recorded. Lastly, cultural transmission is affected by both common descent and spatial proximity, i.e., societies that are closely related and/or geographically close are more likely to show similarities in their cultural repertoires^38–40^. While using the SCCS excludes close dependencies between societies, non-independence of datapoints is not fully alleviated. Hence, we address this issue statistically. Specifically, language phylogenies are routinely used to account for cultural similarity due to shared ancestry^41^. This is because languages are a good representation of the history of expansions and splits of cultural groups and thus represent reasonable estimates of the population history. We use a recent phylogeny of languages^42^ (Supplementary Fig. 6) to compute a matrix of linguistic distances between societies. Further, we use the latitude/longitude coordinates of societies to compute a matrix of spatial distances. We include both these matrices in our models to account for spatial proximity and common descent (see methods for full details).

The number of functions dogs fill in a society was positively associated with positive care and personhood, and negatively associated with negative treatment or dogs (Fig. 2, Supplementary Table 1). These findings show that keeping dogs for multiple purposes results in closer dog-human relationships. When looking at the presence and absence of each function separately (hunting, defence, guarding herds, herding, and carry, Fig. 3, Supplementary Table 2), we find that the odds of dogs given positive care show a substantial increase when dogs are used for herding. We note that guarding herds and carry also show sizeable impacts on positive care (> 80% of coefficient values for these functions are above 0), but hunting and defence show minuscule effects. The odds of dogs being given negative treatment decrease with the presence of all functions except guarding herds; however, these effects are substantial only for the function of defence. Lastly, the odds of dog personhood increase substantially in societies that use dogs for hunting.

### Treatment of dogs, subsistence, ecology, and nuisance parameters

We find that the odds of positive care and personhood are lowered by high levels of animal husbandry (which coincide with pastoral societies that predominantly use big domestic animals, Supplementary Fig. 5). Interestingly, mean annual temperature increases odds of positive care. Conversely, temperature has been previously linked with low mutual dog–human utility^33^, however, function and the characteristics of dog-human relationships are separated in our models (as opposed to^33^). As expected, we find that the odds of positive care, negative treatment, and personhood generally increase with higher number of paragraphs about dogs in ethnographies. In all these models, the variance associated with linguistic clustering is smaller than the variance associated with spatial clustering. Thus, spatial dependencies are stronger than phylogenetic clustering in our sample (Supplementary Tables 1, 2). Lastly, we look at how well the data fit our models, i.e., the proportion of variance in positive care, negative treatment, and personhood that is explained by our predictors. Overall, the predictors explained between 61–75% of variance in response variables (when considering number of functions, Supplementary Table 1), and between 79 and 87% when considering each function individually (Supplementary Table 2). We note that, in general, around half of the variance in positive care, negative treatment, and personhood was predicted by phylogenetic and spatial dependencies (see methods for details, Supplementary Tables 1, 2).

## Discussion

Here we investigate the relationship between dog functions and the characteristics of dog-human bonds in a global sample. We predicted that dog-human relationships are closer (increased positive care and personhood, and decreased negative treatment) when dogs fill more roles in societies, and when these roles require a high investment and/or intense cooperation between dogs and humans. Indeed, we find that dog-human interactions are closer in societies with increased number of functions. We also find increased positive care in societies that keep herding dogs, and an increased odds of treating dogs akin to a person in societies that use dogs for hunting. Our results also offer some unexpected outcomes. Hunting does not associate with increased positive care in our sample. We also see substantial decreases in the odds of negative treatment of dogs in societies that use dogs for defence. This effect comes as a surprise, as we intuitively associated the presence of watchdogs with a distant bond between dogs and their owners. All in all, our results support the proposed link between function and the characteristics of dog-human relationships in a cross-cultural sample.

### Increased affect and utility with multiple dog functions, but uneven effects across individual roles

The drivers behind our behaviour towards non-humans are complex. Serpell^43^ attempts to reduce this complexity to two main axes: affect—people’s emotional perceptions of animals, and utility, people’s perception on the economic value of animals. These axes were primarily developed from studies concerning WEIRD dogs. Nonetheless, they can serve as a scaffold for interpreting drivers of perception and attitudes towards dogs in our worldwide sample as well. We find evidence of increased positive care and decreased negative treatment when dogs are kept for multiple purposes. This result is intuitive, as with multiple roles, dogs likely bring substantial benefits to their owners and are probably perceived by societies as having a high practical value. Multiple functions can also equate with dogs being more present in the day-to-day life of people in these societies, potentially driving a culture of increased affect towards dogs. This line of argument is reflected in our results by the substantial increase in personhood with number of functions. But not all dog roles impact the same on treatment and perception of dogs in societies.

All functions associate with a trend of increased odds of primary caregiving, i.e., we see above 0 median values of posterior coefficients in models. Most roles also tend to decrease odds of negative treatment (except for guarding herds), and increase odds of personhood (except for the role of carry animals). These results further signal an association between a close dog-human relationship in societies where dogs are useful to humans. However, the effect sizes of functions are vastly different, suggesting we need to look beyond explanations based on the net benefits (i.e., usefulness versus investment) of dogs. Such differences could be explained by the amount of cooperation with humans involved in various dog functions. Our results, however, show a nuanced picture.

### No evidence for positive care in societies that keep dogs for hunting

Keeping dogs for herding associate with a substantial increase in odds of positive care (an effect also reflected, albeit more modestly, for guarding herds and the function of carry animal). Surprisingly, however, this is not true for the role of hunting. Intuitively, we would expect dogs to provide great economic benefits in societies that use hunting dogs. Ethnographies indeed record telling observations on the values of hunting dogs (e.g., “We have seen a few [hunting] dogs for which the owner had paid a buffalo”,^44^ on Eastern Toraja). However, the economic value of hunting dogs can vary greatly around the world (e.g., in the Neotropics, the return rates of hunting with dogs differ drastically depending on prey type and population density of prey species,^34^). Net benefits of hunting with dogs can also vary with time (e.g., in !Kung or Semang societies, excerpts in Supplementary Table 3). These uneven economic benefits of hunting with dogs likely weaken the chances of finding a substantial increase in positive care with the function of hunting in a global sample. Further, the role of hunting can contrast directly with the dogs’ needs (e.g. “Warao dogs are consistently ill fed and deliberately mistreated, for a hungry hunting dog is preferred to one that is sated”^45^). Often also, hunting dogs are capable, and consequently left to, fend for themselves (Supplementary Table 3). All these likely contribute to our finding that keeping dogs for hunting does not result in a substantially increased primary care for dogs.

### Differential effects and potentially shifting pressures of utility versus affect

Keeping dogs for hunting and as watchdogs are thought to be early functions following their domestication^4,7,9^, whereas herding, guarding herds and carry are relatively more recent additions. Our results show that the more derived functions have a much stronger effect on positive care in contrast to hunting and defence. These findings could thus signal a shift in time towards increased positive care given to dogs with their breeding as more sophisticated work tools. With the development of roles beyond hunting and defence, the perceived and realized practical value of dogs probably increased. Simultaneously, dogs also became more dependent on their humans, as for example is recorded about the Saami herding dogs: “Lack of total independence renders the dog vulnerable in a revealing manner”^46^. While this hypothesis requires rigorous statistical testing, some ethnographic observations are revealing. For example, Kaska peoples used dogs initially for hunting, and then as carry animals (Supplementary Table 3). It is recorded that “formerly, no particular care was taken of dogs. They slept just like wolves and lay inside and outside the camp at any place”. In the present, however, dogs are fed and provided with brush shelters during winter times^47^. In contrast to positive care, our findings show a substantial increase in personhood odds in societies where dogs are used for hunting, a result that is not mirrored for defence or for the more recently developed functions.

The association between hunting and personhood could be explained by the nature of close daily proximity and interactions between hunting dogs and owners. These dogs are sometimes described as being indispensable and habitual around the household of their owners (Supplementary Table 3). This close contact and connection is durable and can extend over the dog’s entire lifespan (e.g., Aweikoma hunting dogs are described as “always around” their owners, and “following their close connection in life, hunting dogs can be killed when their master dies”^48^). Telling examples speak of hunting dogs being “adopted” into families (Supplementary Table 3). Further, hunting dogs can also share a tight connection and close cooperation with their owners while on the hunt (e.g., in Warao “nearly every adult has his special hunting dog” that is “taken along on trips”^49^). Taken together, these findings could indicate a potential shift in time towards a strictly functional perception of dogs as working tools (increased positive care, but lack of effects in personhood) with the emergence of herding, guarding of herds and carry functions. In contrast, the ancestral function of hunting maintains a link with emotionally close and/or anthropomorphized dog-human bonds. More broadly, our results give support to the proposed separation between the impacts of utility (largely associated with primary caregiving) and affect (associated with personhood) on human behaviour towards animals^22^.

### Decreased negative treatment when dogs are kept for defence

We expected distant dog-human relationships in societies that employ dogs for defence. However, we do not see substantial impacts on the function of defence on positive care or personhood. Moreover, we find a substantial decrease in negative treatment in societies that use dogs for defence. We interpret this surprising result in the light of several non-exclusive explanations. Defence roles do not involve close cooperation or close day to day proximity between dogs and humans, and accordingly, we see no effects of keeping dogs for defence on personhood or primary caregiving. These results could suggest a culture of general lack of interest towards defence dogs: watchdogs are often left to roam, scavenge, they are likely more independent and less trained (Supplementary Table 3). Thus, the costs associated with keeping a dog for defence purposes (and for example the need of culling puppies, a variable that is part of our coding of negative treatment) are small. An absence of close working relationships with defence dogs can also translate into an absence of punishment for mistakes (as opposed to other roles, e.g., [the Saami herding dog] “will hear profuse swearing when he does not cease a maneuver or heed a change in instruction”; “not only when the dog has failed his master in some way, but also when the herder needs a scapegoat, the dog may be kicked or beaten”^46^). These likely overlapping arguments could drive the association between decreased negative treatment and the function of defence in our analyses. More broadly, this association suggests candidate modifiers of human behaviour towards animals, alongside affect and utility. Namely, the overall degree of importance given to certain roles (such as defence), as well as the minimum requirements to successfully execute a function (similar to the case of hunting dogs) seem to mediate our expectations regarding how humans treat their animals.

### Distant dog-human relationships with reliance on animal husbandry

We investigated the role of function on dog-human relationships while also accounting for potential covariates. One of these is animal husbandry, and we find that in general, heavier reliance on animal husbandry is associated with less positive care and personhood. It has been proposed that perhaps societies that rely on animal husbandry mainly employ dogs to guard the livestock. Such a role removes dogs from close proximity with humans and could drive distant dog-human relationships. We see, however, that the effects of animal husbandry on dog perception and treatment remain substantial when including dog functions (i.e., accounting for the role of guarding herds) in the models. Moreover, we find that keeping dogs to guard herds shows a trend of increased positive care (expected, given these can be high investment dogs). Thus, our results do not support an explanation based on the role of guarding herds for the impacts of animal husbandry on dog-human relationships. An alternative explanation could be the fact that a reliance on animal husbandry funnels resources associated with animals towards livestock in detriment of dogs. Indeed, in our sample, a heavier reliance on animal husbandry overlaps with the use of predominantly camelids, deer, and bovine, i.e., costly livestock, which would decrease the possibilities to invest in dogs. Further, our findings are in line with the proposed decreased affect towards non-livestock animals (e.g., dogs) when societies largely rely on consumption or the coercive use of animals^43^. More broadly, these results suggest that primary caregiving can vary among the animals that humans keep. However, affect-related behaviours might extend across all animal species present in a society, regardless of their predominant purpose. These arguments set exciting hypotheses for future tests.

### Complexities in dog-human interactions

Ethnographers remark on examples of ambivalent emotions and behaviours towards dogs. For example, Mbuti hunting dogs “valuable as they are, get kicked around mercilessly from the day they are born to the day they die”^50^. The Tuareg “may be cruel to their dogs”, but “they acknowledge that they [dogs] are very wise and intelligent”^51^. Our results show that such examples are widespread, as we find that positive care and negative treatment are not mutually exclusive (both present in 32/77 societies with full data). As Gray and Young^22^ also suggest, these findings show that dog-human relationships reflect a practical balance between primary caregiving and minimizing costs of keeping dogs. That is, baseline positive care is offered so that dogs’ functional purpose is not compromised. If that is achieved, humans then try to cut down costs of keeping the animals, for example, by not providing healthcare or by culls. Our data also highlight the presence of complexities in human behaviour towards dogs at finer levels than recorded by our positive care, negative treatment, and personhood categories. For example, in Barama River Carib society, dogs are generally kept in poor conditions, but they are provided health care (Supplementary Table 3). We could thus expect more nuance in the associations between function and dog-human relationships. However, we did not have enough data entries to investigate how dogs’ roles separately affect each component of the overall positive care, negative treatment, and personhood dimensions.

We note that, generally, our data cannot tell us if, in societies in which dogs fill multiple roles, different dogs are kept for different purposes. Similarly, we have sporadic information on dog breeds. Therefore, we cannot know whether our positive care, negative treatment, and personhood scores characterize all or a restricted part of dogs in a given society. For example, Mutair peoples keep watch dogs that are not allowed indoors, are fed little, and are generally regarded as unclean. In contrast, the salúqis dogs in the same society are particularly well-fed, sleep inside, and are viewed as clean (Supplementary Table 3). These detailed accounts of separate behaviours towards different kind of dogs are not common, but they remind us that while a society can show the presence of positive care for example, this behaviour can affect different dogs in different ways. Likewise, our results do not incorporate intra-societal variation in dog-human bonds, i.e., whether dog treatment differs across children versus adults, men versus women (discussed in^33^), servants versus owners etc.

### Dog functions and dog-human relationships: cultural and psychological perspectives

Dogs and humans have a special relationship that manifests in an extraordinary array of psychological and physiological changes that affect both dogs and their owners. Studies done on WEIRD dogs show that the function of dogs can impact on dog behaviour, dog-owner relationship and on dogs’ communication and problem-solving skills^14,15,17–20^. Further, how dogs are perceived in a society impacts on our own ability to understand (and presumably communicate with) dogs^52^. Here we show how the purpose for which dogs are kept and the characteristics of dog-human relationships (positive care, negative treatment, and personhood) are closely linked in a sample of 124 globally distributed societies. Our findings also show that different functions affect treatment of dogs differently. For example, while herding (and to a lesser extent, guarding of herds and carry functions) associates with increased positive caregiving, hunting results in increased odds of dog personhood. By tying together findings from anthropology, biology, and psychology, we open the possibility of cultural correlates that disrupt the assumed universality of dog skills. Specifically, our results set theoretical and empirical foundations for testing whether the behaviour and social-cognitive skills of dogs are impacted by the societies dogs live in. Hunting dogs associate with a culture of anthropomorphized dog-human interactions, much like the owner-pet relationship in WEIRD societies today. Do we then expect that generally, hunting dogs show most exceptional capacities to understand, communicate, and cooperate with humans? Or would that be true of the well-treated herding dogs, likely a more sophisticated and recent function? Do we expect modest dog-owner communication when watch dogs are tested? These hypotheses represent exiting next steps into understanding the universal versus culture-dependent characteristics of our bond with our best friends.

## Methods

### Data collection: eHRAF search and coding of variables

We started the search procedure on eHRAF’s “Advanced search” page (https://ehrafworldcultures.yale.edu/ehrafe/booleanSearchSetup.do?forward=booleanForm). We narrowed the search by: (i) culture, (ii) OCM topic (domesticated animals—subject code 231), and (iii) keywords (dog* hound* canine* mongrel* mutt* pup*, with “OR” as delimitation between subjects and keywords). We checked and saved the matching paragraphs. Each variable of interest is coded as: 0 (absent, used if there was enough info, i.e., at least more than two paragraphs, to evaluate that a specific function or perception regarding dogs is not present in that society), 1 (present, but rare), 2 (present, but no information regarding the frequency), 3 (present, and common), and NA (missing information, i.e., insufficient information in the documents to derive a coding for the variable). For our analyses, we collapsed the codes for each variable into simple occurrence, i.e., absence (initial code = 0), presence (initial codes = 1, 2, or 3), and NA (missing information).

### Function and treatment of dogs

For each of the 124 societies (cultures) in our sample, we coded the presence or absence of the following functions: (i) hunting (actively track, chase, flush prey), (ii) defence (dogs used for defence and guarding everything except herds), (iii) guarding herds, (iv) herding, (v) pulling the sled, (vi) as pack animals, (vii) keeping dogs as playthings, (viii) using dogs as a status symbol, (ix) waste/vermin removal, (x) assistance, (xi) rescue, and (xii) using dogs for food. We also recoded the presence of stray (i.e., unowned) dogs. We merged the codes for pack animals and for pulling the sled into one overall function representing whether dogs are used for carrying. For each society, the code for the new variable was set to: (i) NA, if both functions were recorded as NAs, (ii) absent, if both functions were recorded as absent, and (iii) present—if one or both functions were scored as present. Further, we code variables associated with three dimensions of the dog-human relationship: positive care, negative treatment, and personhood. A society is scored as having positive care if any of the following variables are recorded as present in the ethnographies: dogs are fed with human food, breastfed, allowed to sleep indoor, allowed indoors, groomed, played with, trained, provided with puppy care (intentional raising of puppies), provided with healthcare, or provided with special equipment designed for their well-being. Societies are recorded as showing negative treatment towards their dogs if dogs are not fed, not provided healthcare, physically abused, or killed as part of regular culling acts, in disagreement with other humans, or simply as a result of aggression towards dogs. We do not consider the cases when dogs are killed as part of ritual acts, or when dogs are killed because they are very old/diseased. Lastly, societies are recorded as attributing personhood to their dogs if dogs are given names, talked to, buried, mourned, or treated as kin or close friends, in distinction to other animals. In all societies save one, we do not have any records by which personhood variables could be coded as absent (i.e., the societies either show the presence of these variables, or there is no information available). The only exception to this rule is seen in Manus, who do not talk or give names to their dogs. For personhood variables thus it is most plausible that an absence of information in ethnographies translates into an absence of behaviour. Given this note, we equate NA personhood codes to 0.

### Covariates for the treatment of dogs

Alongside dog roles, we include annual mean temperature and subsistence strategy (farming propensity and animal husbandry), variables previously shown to affect dog’s general utility to humans^33^. We used the Baseline Historical (1900–1949) CCSM ecoclimate model dataset^53^ to extract the mean value of monthly temperatures across the year at the locations of societies in our dataset. We access the climatic data via D-PLACE (https://d-place.org/about, variable name in D-PLACE: AnnualMeanTemperature).

We follow Vilela et al.^54^ to obtain a metric reflecting dependence on agriculture, as opposed to hunting, gathering, and fishing (metric labelled as farming propensity in^54^). Specifically, for each society, we used the Ethnographic Atlas^55,56^ dataset as available via D-PLACE to extract data on dependence on agriculture (variable code EA005), hunting (EA002), gathering (EA001), and fishing (EA003) in the form of percentage intervals. For example, !Kung shows 76–85% dependence on gathering, 16–25% dependence on hunting, 0–5% dependable on fishing, and 0–5% dependence on agriculture. We first sample a random integer number from each interval to code the subsistence of each society. Subsistence strategy patterns are not independent from each other, hence we run a principal component analysis (PCA) on these variables, a technique commonly used to reduce dimensionality. We do so by using the function princomp() from the R package *compositions*^57^. In our dataset, principal component one (PC1) reflects a continuum that separates an agricultural subsistence mode from hunting, gathering, and fishing (Supplementary Fig. 4). To account for variation within each interval (i.e., a 0–5% dependence on fishing could reflect any integer in that interval), we repeat the sampling procedure and the associated PCA 500 times. Across these runs, agriculture loads substantially on PC1 (mean loadings = 0.852, standard deviation = 0.003), and PC1 captures on average 63% variation in subsistence modes (standard deviation = 0.008). For each society, we averaged the values for PC1 across our 500 runs to compute the final value of farming propensity. We further used the D-PLACE variable EA004, to characterize societies’ reliance on animal husbandry (Supplementary Fig. 5). Similar to EA001–EA003, and EA005, this variable contains percentage intervals, and so we considered the mean value to reflect animal husbandry dependence in each society (e.g., !Kung shows a dependence on animal husbandry of 0–5%, and we considered a value of 2.5 for this society). Lastly, we also include the number of paragraphs that record information about dogs in ethnographies, as with more paragraphs we expect to see a higher likelihood of rare behaviours being recorded, and thus a higher chance of false positive associations between dog functions and treatment of dogs.

### Phylogenetic and spatial dependence

We account for common descent and spatial dependencies by including two matrices as covariates in our models. First, we build a variance–covariance matrix from a global language phylogeny proposed by Jäger^42^. We prune the initial 7432 tip languages in the phylogeny to 114 societies common to our dataset and the phylogeny (Supplementary Fig. 6). We then use vcv() function in the R package *ape*^58^ to estimate the variance–covariance matrix, which captures the similarity in tips due to their shared descent on the off-diagonal. We account for cultural similarities due to spatial proximity by building a spatial covariance matrix based on the longitude and latitude coordinates for each society, and using the function varcov.spatial() in the R package *geoR*. We set the smoothness and variance parameters to 1.

### Statistical modelling

We run separate models for estimating the predictors of positive care, negative treatment, and dog personhood. We use the package *brms*^60^ in the R software^59^ to run binomial Bayesian models, setting the family distribution for the response variable to Bernoulli, and an exponential prior for the group-level effects (i.e., for the phylogenetic and spatial clustering). When looking at the predictors for how societies threat their dogs, we prune our dataset to 97 societies for which we had information on all main functions (hunting, defence, guarding herds, herding, and carry animals). These numbers are then further pruned for societies with data on all other predictors, phylogenetic information, and data on the response variables. The final datasets for testing predictors of dog-human relationships contained: n = 80 societies for positive care, n = 61 societies for negative treatment, and n = 81 societies for personhood. The differences in sample size between these datasets are attributed to the differences in number of societies with full data for positive care, negative treatment, and personhood, respectively. We do not summarize the posterior distributions of coefficient estimates, rather we plot these in entirety. We consider a predictor to have a substantial impact on the odds of the response variable if 95% or more of the coefficient estimate posterior distribution is below 0 (for decreased odds) or above 0 (for increased odds). We compute model fit using a Bayesian alternative to standard R-squared^61^. In a non-Bayesian context, the R-squared is defined as the variance of the predicted values divided by the variance of the data. In a Bayesian model, the numerator can be larger than the denominator in that equation. Hence, we use the proposed Bayesian alternative, i.e., the variance of the predicted values divided by the variance of predicted values plus the expected variance of the errors^61^. We compute both marginal (variance explained by fixed effects) and conditional (variance explained by fixed effects, phylogenetic, and spatial effects) R-squared values using the function r2_bayes() in *rstan*^62^ (Supplementary Tables 1, 2).

## Supplementary Information

Supplementary Information 1.

Supplementary Information 2.

Supplementary Information 3.

Supplementary Information 4.

Supplementary Information 5.

Supplementary Information 6.

Supplementary Information 7.

## Data Availability

The code for performing the models and all data files have been uploaded as supplementary data files.
