# Supplementary material for: Function predicts how people treat their dogs in a global sample
Source: Sci Rep. 2023 Mar 27;13:4954. doi: 10.1038/s41598-023-31938-5 (PMC10042878; doi:10.1038/s41598-023-31938-5)
Supplement: Supplementary file 1 — Supplementary Information 1. [file 41598_2023_31938_MOESM1_ESM.doc]

**Supplementary Data – separate files**

Supplementary Data1 (separate .xslx file). Raw data for 124 societies on dog function and dog-human relationships.

Supplementary Data2 (separate .csv file). Predictors for the dimensions of dog-human relationships.

Supplementary Data3 (separate .zip file). Phylogeny of languages used in our analyses.

Supplementary Data 4 (separate .zip file). Code files to run the brms models.
