# Supplementary material for: Function predicts how people treat their dogs in a global sample
Source: Sci Rep. 2023 Mar 27;13:4954. doi: 10.1038/s41598-023-31938-5 (PMC10042878; doi:10.1038/s41598-023-31938-5)
Supplement: Supplementary file 2 — Supplementary Information 2. [file 41598_2023_31938_MOESM2_ESM.docx]

**Supplementary tables**

**Supplementary Table 1**. Summary of models predicting the characteristics of dog-human relationships. For each parameter, bulk ESS and tail ESS are effective sample size measures, and Rhat is the potential scale reduction factor on split chains (at convergence, Rhat = 1). The median and midspread of coefficient estimates are computed considering the entirety of the posterior values. The midspread is computed following the formula: midspread = median (values) +/- iqr (values) * 1.35, where iqr is the interquantile range. Two estimates of model fit are reported: marginal variance explained by fixed effects) and conditional R-squared (variance explained by fixed effects, phylogenetic, and spatial effects).

| A Response variable = positive care | | | | | |
| --- | --- | --- | --- | --- | --- |
| Population-Level Effects | | | | | |
|  | Median coefficient estimate | Midspread | Rhat | Bulk ESS | Tail ESS |
| Intercept | 1.46 | -0.40: 3.33 | 1 | 1313 | 1240 |
| Number of functions | 1.53 | 0.31: 2.74 | 1 | 1371 | 1208 |
| Farming propensity | -0.22 | -1.01: 0.55 | 1 | 1207 | 1394 |
| Mean annual temperature | 1.66 | 0.52: 2.79 | 1 | 1379 | 1247 |
| Animal husbandry | -1.59 | -2.70: 0.47 | 1 | 1341 | 1352 |
| Number of paragraphs | 3.85 | 1.48: 6.22 | 1 | 1333 | 1352 |
| Group-Level Effects | | | | | |
| Phylogenetic effects: SD = 1.53  Spatial effects: SD = 2.20 | | | | | |
| Conditional R^2^ = 0.61  Marginal R^2^ = 0.28 | | | | | |

| B Response variable = negative treatment | | | | | |
| --- | --- | --- | --- | --- | --- |
| Population-Level Effects | | | | | |
|  | Median coefficient estimate | Midspread | Rhat | Bulk ESS | Tail ESS |
| Intercept | 4.88 | 1.42: 8.34 | 1 | 1368 | 1323 |
| Number of functions | -2.23 | -3.91: -0.55 | 1 | 1359 | 1431 |
| Farming propensity | 0.03 | -1.11: -1.16 | 1 | 1434 | 1170 |
| Mean annual temperature | -1.55 | -3.12: -0.00 | 1 | 1302 | 1352 |
| Animal husbandry | 1.25 | -0.23: -2.74 | 1 | 1381 | 1249 |
| Number of paragraphs | 2.81 | 0.68: 4.94 | 1 | 1240 | 1313 |
| Group-Level Effects | | | | | |
| Phylogenetic effects: SD = 1.09  Spatial effects: SD = 5.53 | | | | | |
| Conditional R^2^ = 0.74  Marginal R^2^ = 0.32 | | | | | |

| C Response variable = personhood | | | | | |
| --- | --- | --- | --- | --- | --- |
| Population-Level Effects | | | | | |
|  | Median coefficient estimate | Midspread | Rhat | Bulk ESS | Tail ESS |
| Intercept | -3.00 | -5.75: -0.24 | 1 | 1228 | 1433 |
| Number of functions | 2.56 | 0.84: 4.27 | 1 | 1142 | 1360 |
| Farming propensity | -1.19 | -2.34: -0.02 | 1 | 1424 | 1289 |
| Mean annual temperature | 0.04 | -0.99: 1.08 | 1 | 1315 | 1301 |
| Animal husbandry | -1.65 | -3.05: -0.23 | 1 | 1138 | 1374 |
| Number of paragraphs | 1.94 | 0.34: 3.54 | 1 | 1286 | 1394 |
| Group-Level Effects | | | | | |
| Phylogenetic effects: SD = 0.97 | | | | | |
| Spatial effects: SD = 5.62 | | | | | |
| Conditional R^2^ = 0.75  Marginal R^2^ = 0.35 | | | | | |

**Supplementary Table 2.** Summary of models predicting the characteristics of dog-human relationships. For each parameter, bulk ESS and tail ESS are effective sample size measures, and Rhat is the potential scale reduction factor on split chains (at convergence, Rhat = 1). The median and midspread of coefficient estimates are computed considering the entirety of the posterior values. The midspread is computed following the formula: midspread = median (values) +/- iqr (values) * 1.35, where iqr is the interquantile range. Two estimates of model fit are reported: marginal variance explained by fixed effects) and conditional R-squared (variance explained by fixed effects, phylogenetic, and spatial effects).

| A Response variable = positive care | | | | | |
| --- | --- | --- | --- | --- | --- |
| Population-Level Effects | | | | | |
|  | Median coefficient estimate | Midspread | Rhat | Bulk ESS | Tail ESS |
| Intercept | 3.92 | -0.22: 8.09 | 1 | 1115 | 991 |
| Hunting | 1.07 | -1.92: 4.08 | 1 | 1473 | 1437 |
| Defense | 0.71 | -1.96: 3.40 | 1 | 1342 | 1349 |
| Guarding herds | 5.41 | -0.49: 11.32 | 1 | 1372 | 1395 |
| Herding | 10.07 | 2.06: 17.19 | 1 | 1378 | 1254 |
| Carry | 5.45 | -1.02: 11.93 | 1 | 1387 | 1433 |
| Mean annual temperature | 4.17 | 1.80: 6.54 | 1 | 1331 | 1391 |
| Farming propensity | -0.19 | -1.70: 1.32 | 1 | 1455 | 1280 |
| Animal husbandry | -3.93 | -6.42: -1.44 | 1 | 1327 | 1308 |
| Number of paragraphs | 7.98 | 3.53: 12.43 | 1 | 1250 | 1185 |
| Group-Level Effects | | | | | |
| Phylogenetic effects: SD = 1.98 | | | | | |
| Spatial effects: SD = 6.46 | | | | | |
| Conditional R^2^ = 0.79  Marginal R^2^ = 0.35 | | | | | |

| B Response variable = negative treatment | | | | | |
| --- | --- | --- | --- | --- | --- |
| Population-Level Effects | | | | | |
|  | Median coefficient estimate | Midspread | Rhat | Bulk ESS | Tail ESS |
| Intercept | 7.39 | 1.62: 13.17 | 1 | 1255 | 1271 |
| Hunting | -1.80 | -6.77: 3.17 | 1 | 1315 | 1258 |
| Defense | -9.72 | -14.37: -5.05 | 1 | 1397 | 1204 |
| Guarding herds | 4.10 | -2.79: 11.01 | 1 | 1255 | 1265 |
| Herding | -1.44 | -7.24: 4.36 | 1 | 1443 | 1394 |
| Carry | 0.23 | -7.01: 7.47 | 1 | 1504 | 1232 |
| Mean annual temperature | -1.90 | -4.50: 0.69 | 1 | 1483 | 1382 |
| Farming propensity | 0.29 | -1.72: 2.30 | 1 | 1179 | 1245 |
| Animal husbandry | 0.49 | -2.38: 3.38 | 1 | 1346 | 1233 |
| Number of paragraphs | 5.10 | 1.64: 8.57 | 1 | 1265 | 1280 |
| Group-Level Effects | | | | | |
| Phylogenetic effects: SD = 1.22 | | | | | |
| Spatial effects: SD = 9.42 | | | | | |
| Conditional R^2^ = 0.85  Marginal R^2^ = 0.39 | | | | | |

| C Response variable = personhood | | | | | |
| --- | --- | --- | --- | --- | --- |
| Population-Level Effects | | | | | |
|  | Median coefficient estimate | Midspread | Rhat | Bulk ESS | Tail ESS |
| Intercept | -13.23 | -20.05: -6.41 | 1 | 1302 | 1354 |
| Hunting | 17.04 | 9.98: 24.11 | 1 | 1264 | 1273 |
| Defense | 0.97 | -2.13: 4.09 | 1 | 1349 | 1228 |
| Guarding herds | 3.05 | -3.83: 9.94 | 1 | 1258 | 1377 |
| Herding | 1.20 | -4.28: 6.69 | 1 | 1295 | 1220 |
| Carry | -3.12 | -10.33: 4.06 | 1 | 1329 | 1394 |
| Mean annual temperature | -2.32 | -4.83: 0.20 | 1 | 1443 | 1436 |
| Farming propensity | -2.11 | -4.14: -0.11 | 1 | 1395 | 1373 |
| Animal husbandry | -2.50 | -5.07: 0.06 | 1 | 1246 | 1391 |
| Number of paragraphs | 1.72 | -0.56: 4.01 | 1 | 1061 | 1283 |
| Group-Level Effects | | | | | |
| Phylogenetic effects: SD = 1.06 | | | | | |
| Spatial effects: SD = 9.78 | | | | | |
| Conditional R^2^ = 0.87  Marginal R^2^ = 0.41 | | | | | |

**Supplementary Table 3.** Relevant details and sections from ethnographies referred to in the main text.

| Source | Relevant section |
| --- | --- |
|  |  |
| Net benefits of hunting dogs vary with time | |
| Lee 1979:142  ^1^ | Dogs are a valuable adjunct in the tracking of all game, large and small, and are especially useful in the killing of steenbok, duiker, warthog, and gemsbok. Their use was widespread in the Dobe area during the 1960s, but apparently much less so in the Nyae Nyae area in the previous decade. John Marshall (1957) noted that dogs were not a significant factor in hunting at /Gausha during his fieldwork in the 1950s. By contrast, at Dobe water hole perhaps a third of the meat taken in 1964 was killed with the aid of dogs, and during one period the proportion rose as high as 75 percent. In 1967–9 dogs appeared to be somewhat less important in hunting than in 1964, suggesting that the use of dogs is situationally variable and therefore not an integral part of the !Kung hunting [Page 143] complex. Nevertheless, when properly trained dogs are available, they can be highly productive. |
| URL: <https://ehrafworldcultures.yale.edu/document?id=fx10-018>. | |
|  | |
| Schebesta and Schütze 1954:64  ^2^ | Nowadays the individual hunt with the blowgun is the only kind of hunting practiced among the Semang. The hunter strolls through the woods, either alone or in the company of one or another companion, often accompanied also by his dog, although the latter is not essential. |
| URL: <https://ehrafworldcultures.yale.edu/document?id=an07-001>. | |
|  | |
| Schebesta and Schütze 1954:106  ^2^ | The Andamanese also prize the dog very much as a hunting animal, although they have had it only since the island was occupied by the British. It has adapted itself very well among them. In the present-day economy of the Semang the dog seems to be unimportant. |
| URL: <https://ehrafworldcultures.yale.edu/document?id=an07-001>. | |
|  |  |
| Schebesta and Schütze 1954:107  ^2^ | There is no doubt that the Semang dogs are excellent hunting dogs, but unfortunately they have no opportunity to hunt. Their hunting consists of roving about in the vicinity of the camp searching for food to satisfy their hunger. |
| URL: <https://ehrafworldcultures.yale.edu/document?id=an07-001>. | |
|  |  |
| Hunting dogs are left to fend for themselves | |
| Adams 1973:16  ^3^ | Scrawny dogs who must scavenge for themselves are kept for hunting. |
| URL: https://ehrafworldcultures.yale.edu/document?id=sr09-002. | |
|  |  |
| Kaska peoples used dogs initially for hunting | |
| Honigmann 1954:37  ^4^ | Before animal traction became prevalent small dogs assisted hunters in running down moose and bear. |
| URL: https://ehrafworldcultures.yale.edu/document?id=nd12-006. | |
|  |  |
| Dogs are indispensable and habitual around the household | |
| Gusinde and Schuetze 1937:286  ^5^ | [Dogs] must be regarded as a constant, indispensable member of the Fuegian family. |
| URL: <https://ehrafworldcultures.yale.edu/document?id=sh06-001>. | |
|  |  |
| Chewings 1936:12  ^6^ | [Dogs] appear to rank with the children in value, and are indulged as bedmates. |
| URL: <https://ehrafworldcultures.yale.edu/document?id=oi08-039>. | |
|  |  |
| Adriani and Kruijt 1951 (V3):366  ^7^ | The dog is the companion of the Toradja. It goes into the house of its master, to whom it is attached. |
| URL: <https://ehrafworldcultures.yale.edu/document?id=og11-004>. | |
|  |  |
| Tocantins and Brunel 1877:15  ^8^ | The women suckle newborn pups at their own breast and shelter them in their hammocks with the children as if they had been born of the same womb. |
| URL: <https://ehrafworldcultures.yale.edu/document?id=sq13-001>. | |
|  |  |
| Hunting dogs can be “adopted” into families | |
| Goodale 1971:154  ^9^ | Women call their dogs by the same kinship terms as they call their own children. Dogs are also given sib membership. |
| URL: https://ehrafworldcultures.yale.edu/document?id=oi20-014. | |
|  |  |
| McIlwraith 1948:174  ^10^ | The old woman and her husband, the childless last survivors of an ancestral family, had transmitted some of their ancestral names to their dog, and had distributed presents to validate the bestowal. |
| URL: <https://ehrafworldcultures.yale.edu/document?id=ne06-001>. | |
|  |  |
| Watchdogs are left to scavenge; they are often untrained | |
| Ebihara 1971:316  ^11^ | They eat, in the words of one villager, “anything and everything,” living on leftovers and whatever else they can find or kill. |
| URL: <https://ehrafworldcultures.yale.edu/document?id=am04-192>. | |
|  |  |
| Koch 1986:73  ^12^ | No one bothers to train them [Kiribati dogs]. |
| URL: <https://ehrafworldcultures.yale.edu/document?id=am04-192>. | |
|  |  |
| Barama River Carib dogs are treated rather poorly, but provided with healthcare | |
| Gillin 1936:44  ^13^ | The children looked on and assisted the dog to be comfortable during the process of parturition. |
| URL: https://ehrafworldcultures.yale.edu/document?id=sr09-001. | |
|  |  |
| Gillin 1936:8  ^13^ | Although the dogs lying around [...] always have a more or less dejected and emaciated appearance, a certain amount of care is taken […] to keep them [the dogs] in good hunting condition. Mosquito worms are dug out of their skin as soon as discovered and chiggers removed from their feet. One often sees a man sitting outside the house examining the feet and skin of his dog. |
| URL: https://ehrafworldcultures.yale.edu/document?id=sr09-001. | |
|  |  |
| Adams 1973:16  ^3^ | Scrawny dogs who must scavenge for themselves are kept for hunting. |
| URL: https://ehrafworldcultures.yale.edu/document?id=sr09-002. | |
|  |  |
| Watchdogs versus salúqis dogs | |
| Dickson 1951:81  ^14^ | The salúqis (pronounced salúqi, plural: salag), on the other hand, are regarded as clean […], and they are allowed to enter tents and sleep in the women's portion at will. |
| URL: <https://ezproxy.shh.mpg.de:20040/document?id=mj04-001>. | |
|  |  |
| Dickson 1951:80  ^14^ | The watch-dog usually sleeps outside the women's apartments and is fed with little bits of rice, bread and dates that are left over from the meals. It is never allowed inside the tents, being unclean (najis). |
| URL: <https://ezproxy.shh.mpg.de:20040/document?id=mj04-001>. | |
|  |  |
| Dickson 1951:377  ^14^ | Shaikh Hamad's saluqis were, however, of a larger and more massive type than those ordinarily found among the tribes of the interior. This may possibly be due to the fact that they did little or no hunting, were fed well and fat rather than muscular. |
| URL: <https://ezproxy.shh.mpg.de:20040/document?id=mj04-001>. | |
|  |  |
|  | |

**Supplementary Table 3 references:**

1. Lee, R. B. *The !Kung San: men, women, and work in a foraging society*. (Cambridge University Press, 1979).

2. Schebesta, P. & Schütze, F. The Negritos of Asia; vol. 2, ethnography of the Negritos: half-vol. 1, economy and sociology. **12**, (1954).

3. Adams, K. J. *The Barama River Caribs of Guyana restudied: forty years of cultural adaptation and population change*. (University Microfilms, 1973).

4. Honigmann, J. J. *The Kaska Indians: an ethnographic reconstruction*. (Yale University Press, 1954).

5. Gusinde, M. & Schütze, F. The Yahgan: the life and thought of the water nomads of Cape Horn. *Die Feuerland-Indianer [The Fuegian Indians]* **II**, (1937).

6. Chewings, C. *Back in the stone age: the Natives of central Australia*. (Angus & Robertson, limited, 1936).

7. Adriani, N. & Kruijt, A. C. *The Bare’e-speaking Toradja of central Celebes (the East Toradja): third volume*. (Noord-Hollandsche Uitgevers Maatschappij, 1951).

8. Tocantins, A. M. G. & Brunel, A. Studies on the Mundurucu Tribe. *Revista trimensal do Instituto Historico, Geographico e Ethnographico do Brazil* **40**, 1–78 (1877).

9. Goodale, J. C. (Jane C. Tiwi wives: a study of the women of Melville Island, North Australia. (1971).

10. McIlwraith, T. F. (Thomas F. *The Bella Coola Indians: volume one*. (University of Toronto Press, 1948).

11. Ebihara, M. *Svay: a Khmer village in Cambodia*. (University Microfilms, 1971).

12. Koch, G. *The material culture of Kiribati*. (Institute of Pacific Studies of the University of the South Pacific, 1986).

13. Gillin, J. *The Barama River Caribs of British Guiana*. vol. 14 (The Museum, 1936).

14. Dickson, H. R. P. *The Arab of the Desert Pbdirect: A Glimpse into Badawin life in Kuwait and Saudi Arabia*. (George Allen & Unwin Ltd, 1951).
