# Supplementary material for: Function predicts how people treat their dogs in a global sample
Source: Sci Rep. 2023 Mar 27;13:4954. doi: 10.1038/s41598-023-31938-5 (PMC10042878; doi:10.1038/s41598-023-31938-5)
Supplement: Supplementary file 3 — Supplementary Information 3. [file 41598_2023_31938_MOESM3_ESM.pdf]

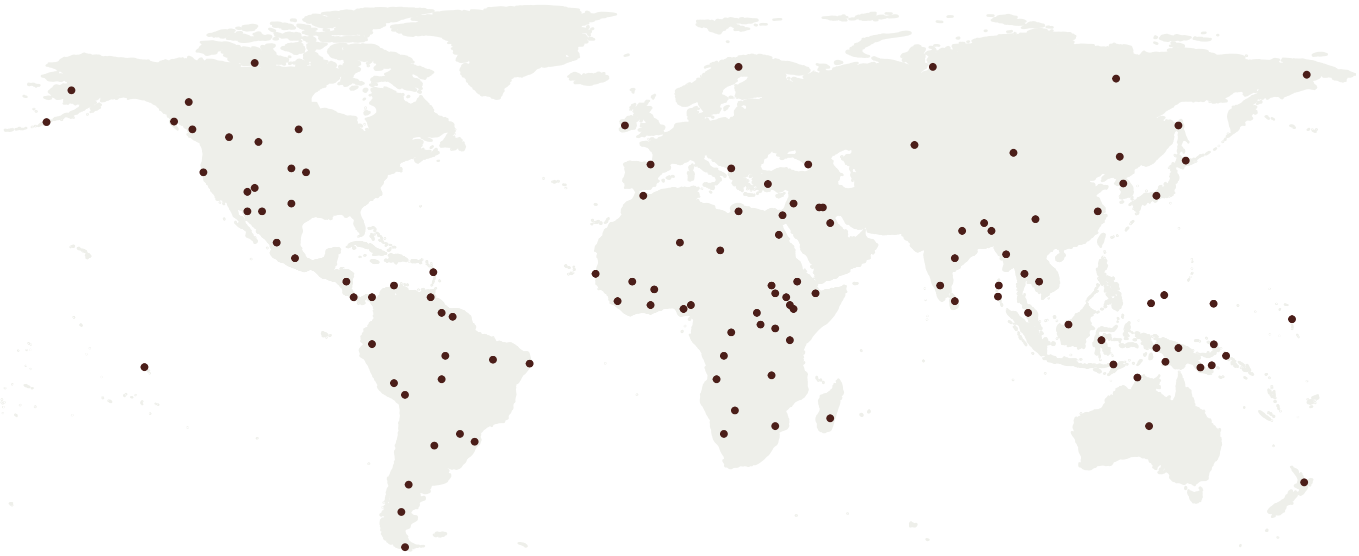

Supplementary Fig. 1 Spatial distribution of the 124 societies considered in our analyses. This map was generated using the function map (database choice: "world") within the *maps* [64] package in the software R [60] (version 4.2.2, https://www.R-project.org/).

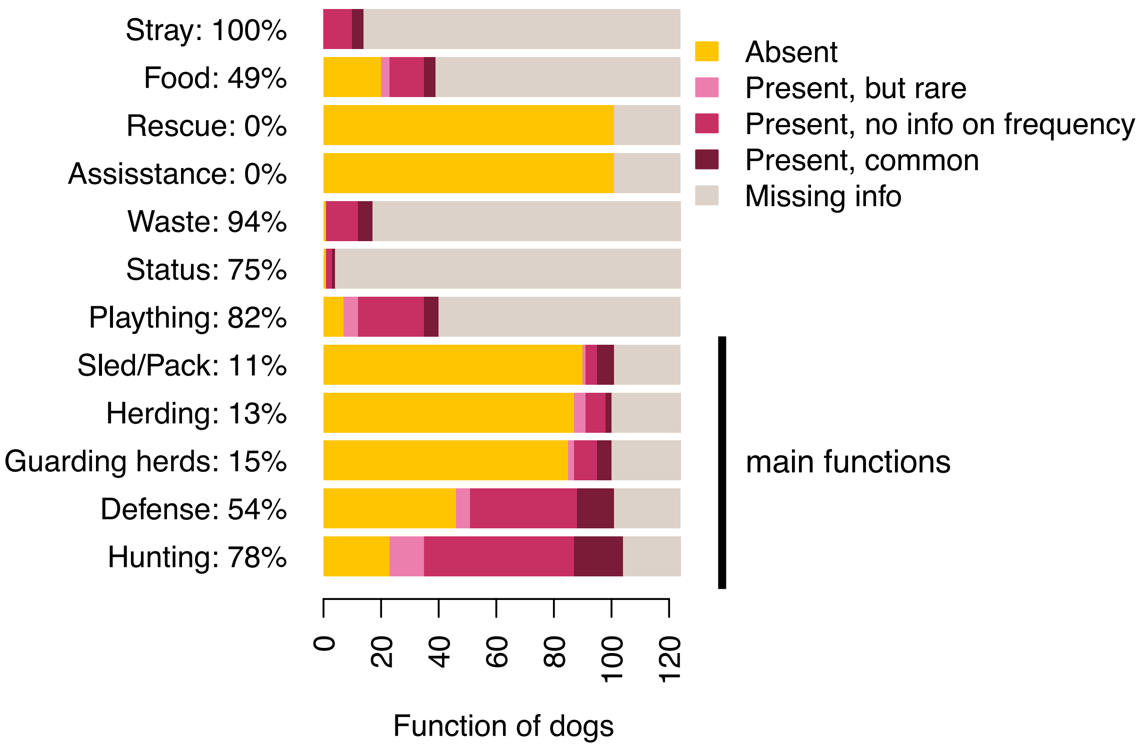

Supplementary Fig. 2 Functions dogs fill in societies. For each function, the percentages show the number of societies with 'present' codes divided by the number of societies with present and absent codes. Functions such as plaything, dogs used for status symbol, for waste/vermin removal, assisstance dogs, rescue dogs, dogs used for food, and stray dogs were excluded from downstream analyses as the codes showed large amounts of missing information, as well as imbalance in the percetege of presence vs absence codes.

Supplementary Fig. 3 Overleaf. Map of (a) positive care, (b) negative treatment, and (c) personhood in our sample. For personhood, all missing information entries are treated as absent in our analyses (detailed in Methods). These maps were generated using the function map (database choice: "world") within the *maps* [64] package in the software R [60] (version 4.2.2, https://www.R-project.org/).

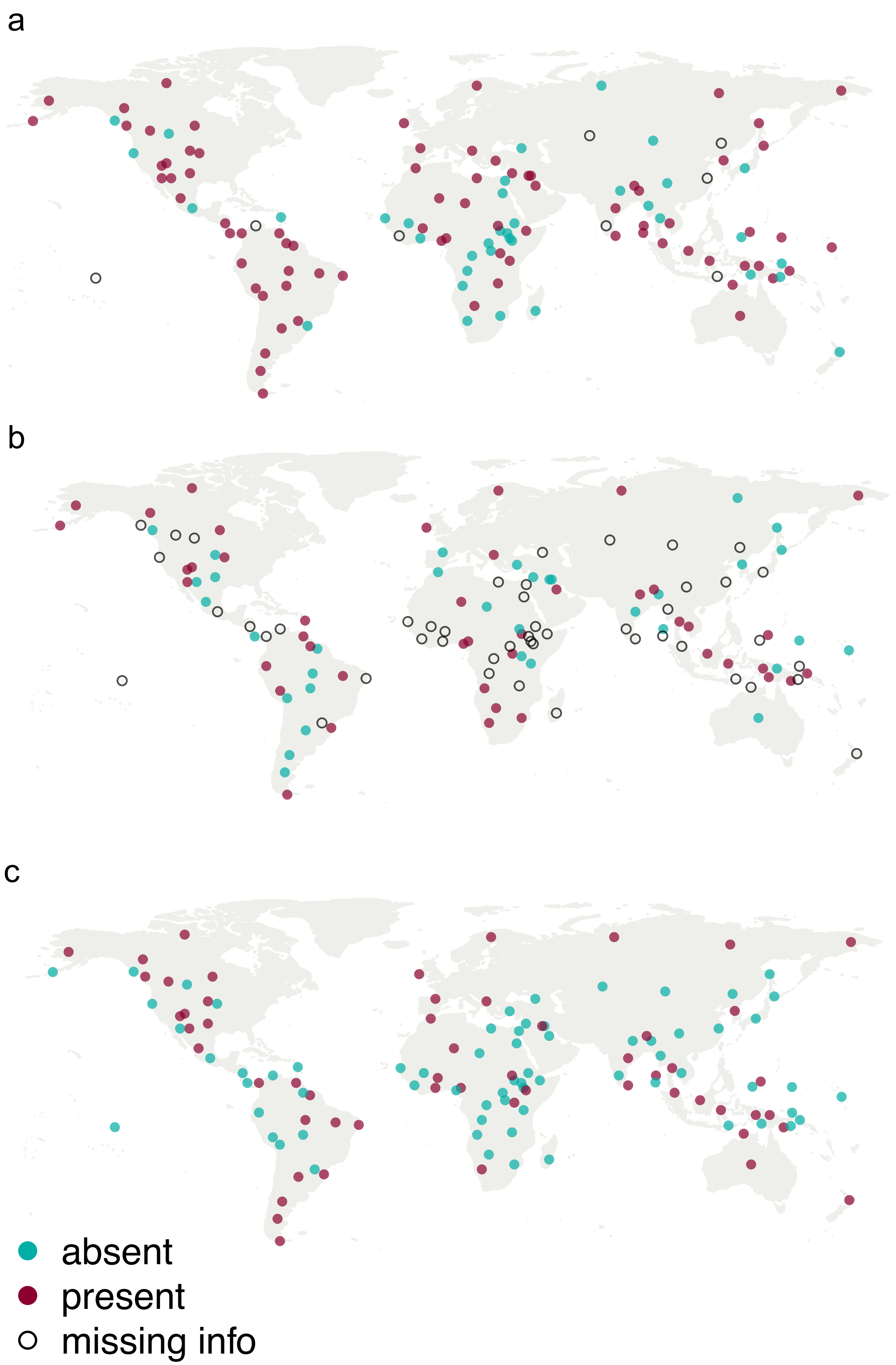
a
b
c
absent
present
missing info

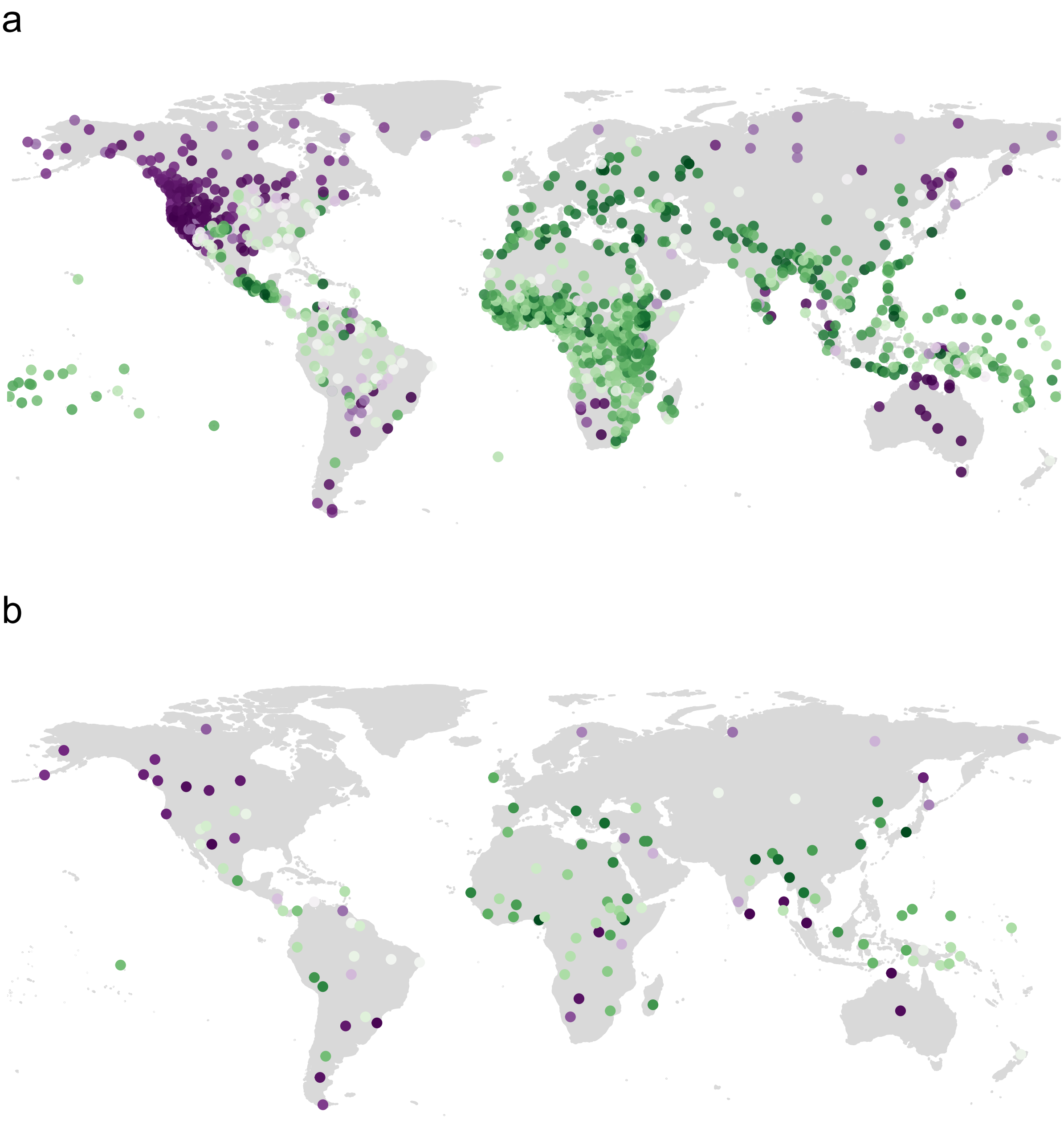

Supplementary Fig. 4 Map of farming propensity, i.e., a principal component describing a continuum from hunting, fishing, gathering (purple end) to agriculture (green end). Panel (a) shows values for all 1291 D-PLACE societies, whereas panel (b) shows our sample. These maps were generated using the function map (database choice: “world”) within the *maps* [64] package in the software R [60] (version 4.2.2, https://www.R-project.org/).

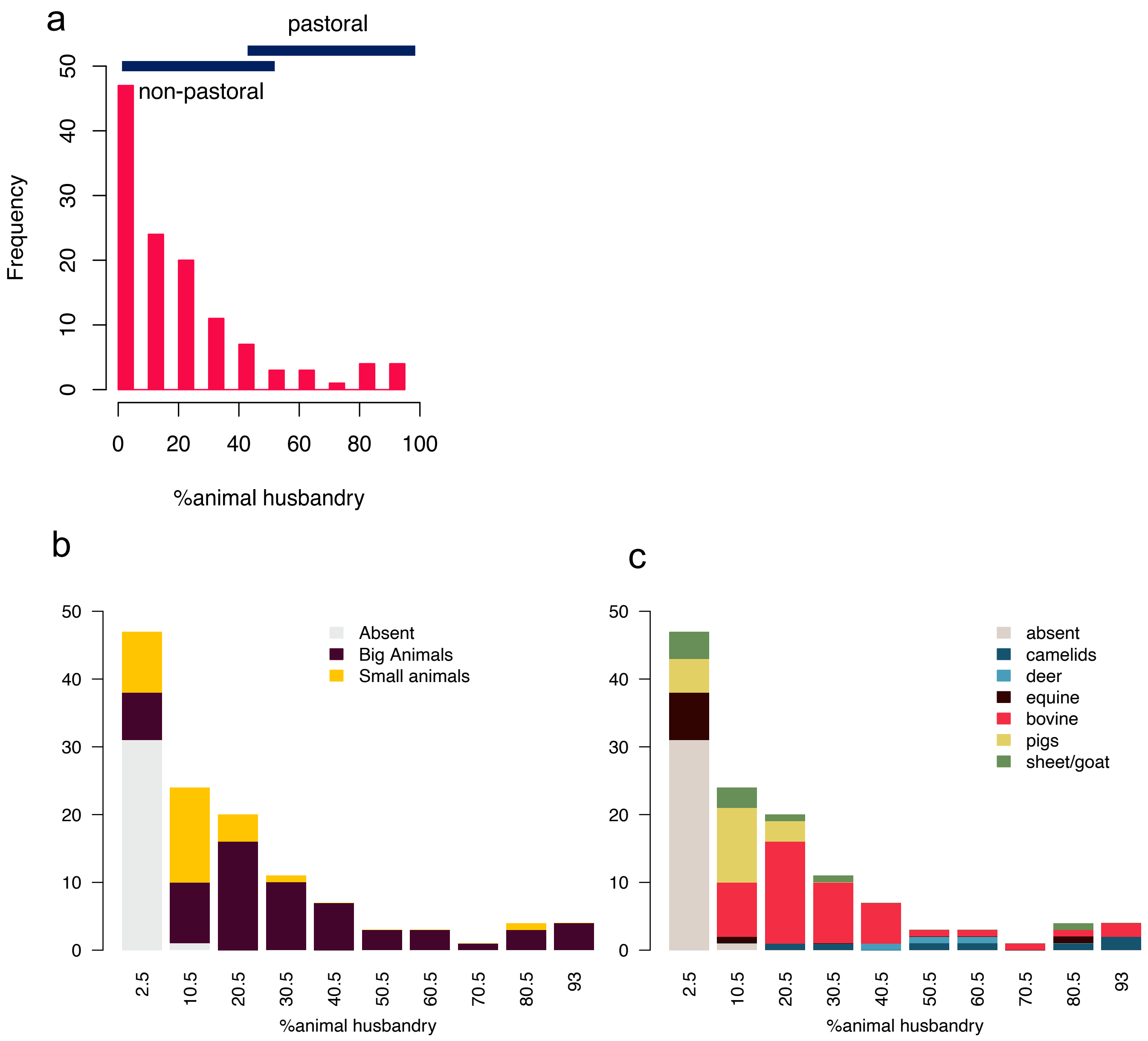

Supplementary Fig. 5 Dependence on animal husbandry in our sample. (a) High values indicate pastoral societies, as defined by the variable EA042 (Subsistence economy: dominant activity) in D-PLACE. Panels (b) and (c) show the dominant animal type for societies grouped in each interval of animal husbandry dependence. Societies that rely on small animals such as pigs, sheep, or goats generally show low values for animal husbandry dependence.

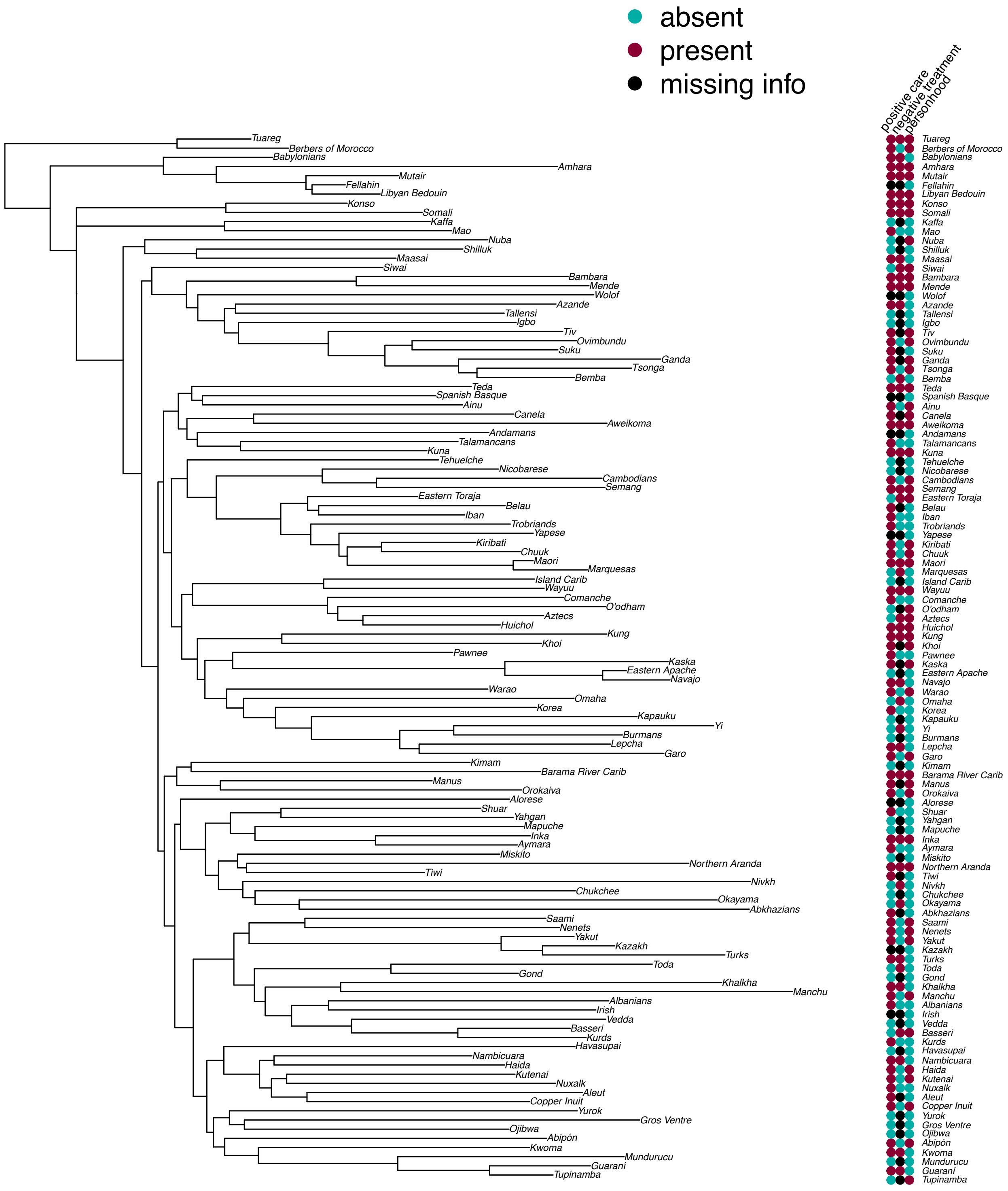

Supplementary Fig. 6 Linguistic phylogenetic hypothesis proposed by Jager (2018) pruned for 114 societies in our dataset for which we could find taxonomic equivalence with the tips included by Jager et al. Coloured circles indicate data coding for positive care, negative treatment, and personhood of dogs.
